# The Burden of General Practice

**Published:** 1908-08-29

**Authors:** 


### The Burden of General Practice

A well-known old print emphasises three phases in the relations between physician and patient during a long illness. In the first tableau the malady seems to have taken a favourable turn, for the leech's figure looms large, and extravagant homage is being paid him, as to a god. Possibly we may think him decidedly lucky to have inspired such sentiments, judging from what has come down to us of mediæval therapeutics. However, by the time of the next scene this gratitude has somewhat cooled. The sick person's friends greet the physician indeed, but they have lost their former veneration; they regard him as a fellow-mortal—as only a man after all. The last shows a further, and a much more abrupt, change for the worse in the lay attitude. For reasons probably not wholly unconnected with the presentation of an account, the doctor is execrated as a downright fiend. Nowadays we have better methods of treatment, but there is still much point in the humour of this old work, humour which perhaps the general practitioner can best appreciate. Invalidism and more or less complete disablement seem to be such trying things that gratitude for slow improvement soon ceases. Even when complete recovery results the physician may be deemed to have done no more than justify his existence; and should the patient die, no doubt many will secretly be of Sancho Panza's opinion that such an unsuccessful doctor is fortunate to receive payment. Not unnaturally the laity look on their medical attendants as people whose business it is to heal ailments of all kinds, and are inclined to favour, in theory at all events, the oft-quoted Chinese method of remuneration, the exact authority for which it would be interesting to learn. The more reasonable and enlightened of our patients are aware that medical therapy is a much more complex undertaking than, say, the repair of a watch; but few appear able to discriminate between different cases of illness as regards relative amenability to treatment. If a man should learn that a friend has got well of much the same sort of symptoms as those which he himself still suffers from, the assurance that they arose from an altogether different and more tractable cause will not be likely to impress him much. Probably, four times out of five, he is wishing in his heart of hearts that he could consult his friend's doctor. Of all such cases the general practitioner sees a good deal. The consultant comes, advises a slight change of treatment, or confirms the previous course—operates or decides that surgery is of no avail. The patient and his friends are cheered, diverted, reassured; at any rate informed authoritatively of what it is important for them to know. Their feelings, if only temporarily, are those of veneration. But it is to their regular medical attendant that fall the tasks of ensuring adherence to regimen after returning health has inspired overconfidence, and of keeping a look out for fresh developments that may involve a total reconsideration of the case; or of breaking bad news, explaining away failure, wisely directing the poignant, pitiable desire of the patient's family for "something to be tried," and finally of consoling and relieving the chronic invalid: in a word, of taking responsible charge of a complicated and uncertain train of events. Whoever can do this with success may rightly be regarded as a man: nor need the judgment be harsh should some occasionally fail.

